# Kidney Transplantation With a Sickle Cell Disease Donor

**DOI:** 10.1016/j.ekir.2020.07.039

**Published:** 2020-08-08

**Authors:** Carole Philipponnet, Julien Aniort, Cyril Garrouste, Jean-Louis Kemeny, Mohammed Hadj-Abdelkader, Anne-Elisabeth Heng

**Affiliations:** 1 Nephrology, Dialysis and Transplantation Department, Clermont Ferrand, University Hospital, Clermont Ferrand, France; 2 UFR Medecine, Clermont Ferrand, France; 3 Anatomy and Pathology Department, Clermont Ferrand, University Hospital, Clermont Ferrand, France

## Introduction

Kidney transplantation is considered as the best treatment for patients with end-stage renal disease. The major factor limiting access to transplantation is the shortage of available kidneys. In the vast majority of cases, deceased sickle cell disease (SCD) patients are excluded from kidney donation because of possible renal damage.

We report a case of kidney transplant recipient with SCD donor and highlight that hemosiderin deposits initially present in epithelial tubular cells disappear few months after exposure to chronic hemolysis is stopped. The patient gave his consent to this publication.

## Case Presentation

**Figure 1:**
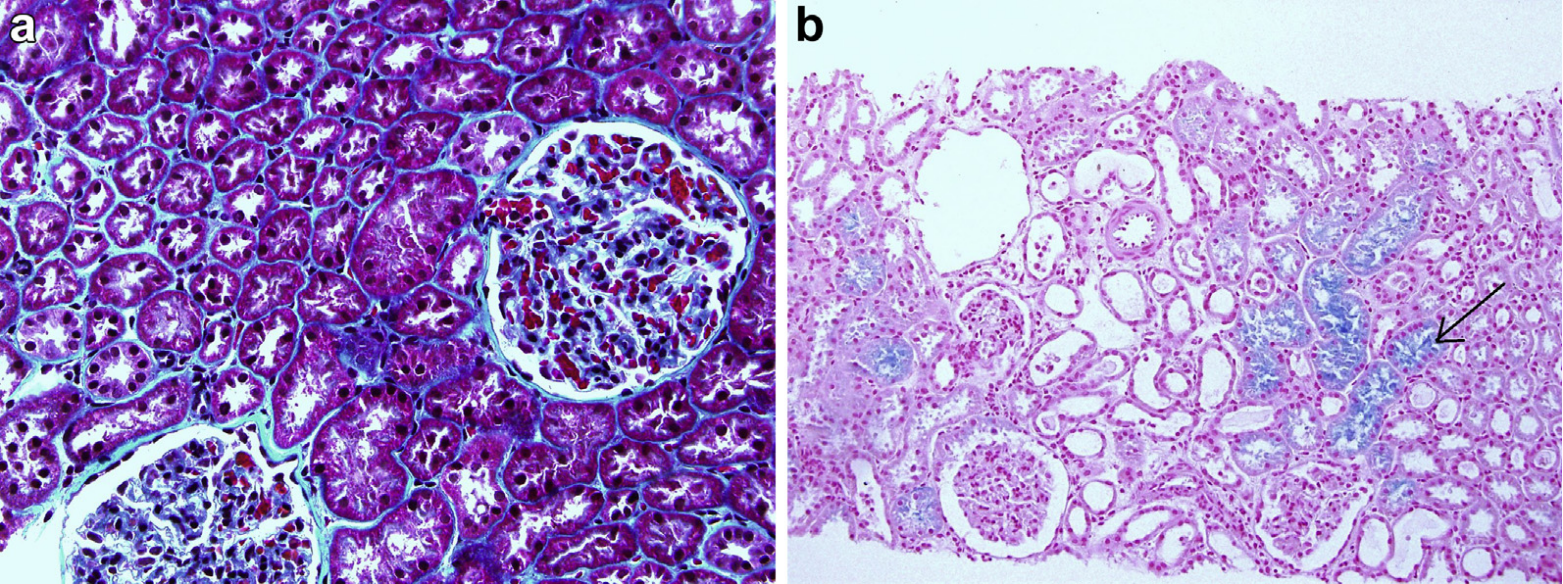
Renal histology (first biopsy). (a) Glomerular enlargement without proliferation, minimal interstitial fibrosis, and no abnormalities in vessels. (Masson trichrome stain; original magnification ×40). (b) Hemosiderin deposits (black arrow) within the proximal tubular cells. (Perls’ Prussian blue stain; original magnification ×20).

**Figure 2:**
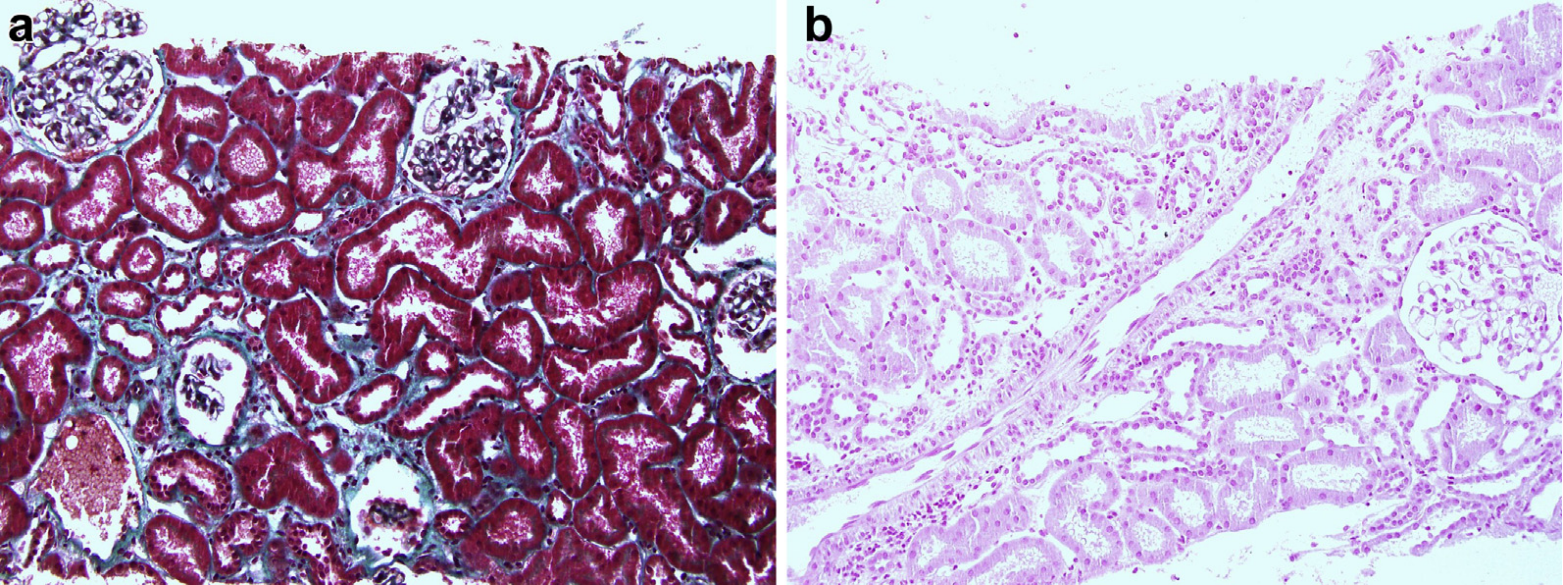
Renal histology (second biopsy). (a) Normal renal histology. (Masson trichrome stain; original magnification ×20). (b) Disappearance of hemosiderosis deposits. (Perls’ Prussian blue stain; original magnification ×20).

A 17-year-old patient was admitted to the nephrology department for his first kidney transplantation. His main medical antecedent was end-stage renal disease requiring chronic hemodialysis secondary to focal and segmental nephropathy. There was no pre-existing immunization to kidney transplantation and no other comorbidities. The donor was a 9-year-old boy with SCD who died as a result of a cerebral vaso-occlusive crisis. He suffered from multiple vaso-occlusive crises since the age of 1 year, and from 3 acute thoracic syndromes having required exsanguinous transfusion. The last crisis was complicated by acute respiratory distress syndrome on acute thoracic syndrome requiring intubation and occlusion of the basilar trunk. There was no acute kidney injury in this patient, the creatinine was steady at 45 μmol/l, eGFR 150 ml/min per 1.73 m^2^ based on the CKD-EPI (Chronic Kidney Disease Epidemiology Collaboration) equation with any proteinuria (0.02 g/l). In order to accept the kidney, a preimplantation wedge biopsy processed as a frozen specimen was obtained (Figure 1). This biopsy showed (i) glomerular enlargement without global segmental sclerosis, no endocapillary hypercellularity, and no thrombi; (ii) minimal interstitial fibrosis; and (iii) no abnormalities in vessels. The only remarkable histologic finding was that of widespread hemosiderosis involving the proximal tubular cells that gave a blue tinge to the specimen on application of Perls Prussian blue stain. Immunofluorescence was negative. The surgery proceeded without complications, with cold ischemia estimated at 34 hours and warm ischemia at 36 minutes. The immunosuppression protocol was as follows: induction by thymoglobulins, mycophenolate mofetil, corticosteroids, and delayed use of calcineurin inhibitors. There was no delayed graft function, and the kidney function remained steady, with creatinine 130 μmol/l, eGFR 80 ml/min per 1.73 m^2^ based on the CKD-EPI equation and proteinuria 0.3 g/d. A protocol kidney biopsy was performed at 3 months (Figure 2). In light microscopy, we observed no signs of rejection (no interstitial inflammation, no glomerulitis, and no capillaritis) and hemosiderin deposits were absent. The immunofluorescence study was negative. One complication occurred during this first year because of a duodenal ulcer. The recipient had a pre-existing iron deficiency at the transplant (ferritin 26 μg/l saturation coefficient 16%). Because of the occurrence of post-transplant anemia and gastric bleeding, iron supplementation was introduced, allowing correction of the deficiency in 2 months.

## Discussion

SCD is one of the most frequent hereditary hematologic disease in the world. Its most severe and common form results from homozygosity for the mutant form of the gene that encodes beta-globin.^1^ Mutant sickle beta-globin results from substitution of valine for glutamic acid at its sixth amino acid. The resulting sickle hemoglobin polymerizes when the concentration of its deoxygenated form exceeds a critical threshold.^1^ The long polymers of deoxygenated sickle hemoglobin distort the red blood cell into the characteristic sickle shape, a process that is exquisitely sensitive to the concentration of sickle hemoglobin within the cell. Sickled red blood cells have greatly reduced deformability, becoming rigid, which hampers their ability to navigate capillaries and very small blood vessels, leading to occlusion of the microvasculature with subsequent pain, ischemia, and organ damage. The pathology of SCD can be directly attributed to sickle hemoglobin polymerization and vaso-occlusion, as well as to secondary effects of chronic hemolysis. In the past few decades, significant advances have been made in our understanding of the complex biochemical and vascular processes that interact with each other to result in vaso-occlusion. Intravascular hemolysis and release of free hemoglobin leading to nitric oxide depletion, endothelial dysfunction, and neutrophil and platelet adhesion are all playing an important contributory role to the pathophysiology of SCD.^1^^,^^2^

Renal involvement contributes to the diminished life expectancy of patients with SCD, accounting for 16% to 18% of mortality.^2^ The spectrum of renal diseases during SCD includes various renal manifestations such as impairment of urinary concentrating ability, defect in urine acidification, renal papillary necrosis, and glomerular injury.^2^^,^^3^ Endothelial dysfunction related to chronic hemolysis and the relative renal hypoxia caused by vaso-occluded sickle red blood cells are probably key factors for the development of renal complications in SCD.^2^ A retrospective study conducted by 14 pediatric nephrology centers has collected the cases of the adolescents and children with SCD who had a renal biopsy performed from January 2000 to December 2015.^4^ Thirty-six SCD patients (ages 4-19 years) were identified; the indications for biopsy were proteinuria (92%) and elevated creatinine (30%). The histopathologic findings were mesangial hypercellularity (75%), focal segmental glomerulosclerosis (30%), membranoproliferative glomerulonephritis (16%), and thrombotic microangiopathy (2%).^4^ Another recent observational study included 1251 black adult patients with sickle cell trait, 230 with sickle cell disease, and 8729 reference patients, with a median follow-up of 8 years.^5^ Sickle cell trait and disease are associated with faster eGFR decline in black patients, with faster decline in sickle cell disease.^5^

African American donor ethnicity is one of 10 risk factors in calculating the kidney donor profile index used in assessing organ quality. A recent study evaluated outcomes of deceased-donor kidney transplants from African American donors stratified by kidney donor profile index. Inferior graft outcomes in recipients of African American kidneys were limited to those with >50% kidney donor profile index, probably because of the effects of risk factors such as APOL1 risk alleles and sickle cell trait.^6^ When efforts are made to replace race with APOL1, some recent data suggest a potential interaction with SCD and sickle cell trait.

SCD is viewed as a contraindication to kidney donation. Two kidney transplant recipients from the same SDC donor were recently reported.^7^ They both have delayed graft function requiring 3 days of hemodialysis post transplantation, but the outcome was favorable. Preimplantation biopsy was performed, and there was no evidence of glomerular, vascular, or interstitial abnormality.^7^ The only abnormality seen is the hemosiderin deposits within some of the tubular epithelial cells.^7^ Intravascular hemolysis is well known to cause kidney injury by several mechanisms.^8^ Indeed, intravascular destruction of red blood cells and the resulting accumulation of hemoproteins can induce oxidative stress and cytotoxicity pathways.^8^

In 2019, in France, 3641 kidney transplants were performed, including 508 from living donors. The waiting time on the kidney transplant list is currently estimated between 2 and 5 years. In view of this shortage of organs, it was decided to transplant from the kidney of a donor with SCD, which is usually excluded from the donation. This decision was adopted in particular in view of the donor's youth and the absence of a history of kidney injury. In order to support this decision, a preimplantation renal biopsy was requested and eliminated damage to the renal parenchyma. Perls staining was subsequently carried out, because there are reports in literature about an accumulation of iron within the tubular cells of SCD patients.

Of great interest, we were able to obtain a protocol biopsy to assess if there was residual donor-derived hemosiderin or transplant damage. We report the disappearance of hemosiderin deposits in kidney transplant recipient. It is important to note that there were no glomerular or vascular lesions in the preimplantation biopsy, and despite the hemosiderin deposits there was no interstitial fibrosis or tubular atrophy. Isolated hemosiderin deposits are a reversible condition after stopping exposure to chronic hemolysis. Like the 2 previous patients reported, the outcome was favorable. It is important to emphasize that the donors in theses cases were children or young adult.

Kidney transplantation with SCD donors seems to be safe with acceptable outcome after a careful examination of the donor’s clinical history and preimplantation biopsy (Table 1).

**Table 1:** Teaching points

• The spectrum of renal diseases during SCD includes various renal manifestations such as impairment of urinary concentrating ability, defect in urine acidification, renal papillary necrosis, and glomerular injury.
• Isolated hemosiderin deposits are reversible after stopping exposure to chronic hemolysis.
• Kidney transplantation with SCD donors seems to be safe, with acceptable outcome after a careful examination of the donor’s clinical history and preimplantation biopsy.

SCD, sickle cell disease.

## Disclosure

All the authors declared no competing interests.
